# Transcriptomic analysis identifies novel candidates in cardiorenal pathology mediated by chronic peritoneal dialysis

**DOI:** 10.1038/s41598-023-36647-7

**Published:** 2023-06-21

**Authors:** Victoria L. Nasci, Pengyuan Liu, Amanda M. Marks, Adaysha C. Williams, Alison J. Kriegel

**Affiliations:** 1 Department of Physiology, Medical College of Wisconsin, 8701 Watertown Plank Road, Milwaukee, WI 53226 USA; 2 Division of Nephrology and Hypertension, Vanderbilt University Medical Center, Nashville, TN USA; 3 Cardiovascular Center, Medical College of Wisconsin, Milwaukee, WI USA; 4 Department of Pediatrics, Medical College of Wisconsin, Milwaukee, WI USA; 5 Center of Systems Molecular Medicine, Medical College of Wisconsin, Milwaukee, WI USA

**Keywords:** Kidney diseases, Renal replacement therapy, Transcriptomics

## Abstract

Peritoneal dialysis (PD) is associated with increased cardiovascular (CV) risk. Studies of PD-related CV pathology in animal models are lacking despite the clinical importance. Here we introduce the phenotypic evaluation of a rat model of cardiorenal syndrome in response to chronic PD, complemented by a rich transcriptomic dataset detailing chronic PD-induced changes in left ventricle (LV) and kidney tissues. This study aims to determine how PD alters CV parameters and risk factors while identifying pathways for potential therapeutic targets. Sprague Dawley rats underwent Sham or 5/6 nephrectomy (5/6Nx) at 10 weeks of age. Six weeks later an abdominal dialysis catheter was placed in all rats before random assignment to Control or PD (3 daily 1-h exchanges) groups for 8 days. Renal and LV pathology and transcriptomic analysis was performed. The PD regimen reduced circulating levels of BUN in 5/6Nx, indicating dialysis efficacy. PD did not alter blood pressure or cardiovascular function in Sham or 5/6Nx rats, though it attenuated cardiac hypertrophy. Importantly PD increased serum triglycerides in 5/6Nx rats. Furthermore, transcriptomic analysis revealed that PD induced numerous changed transcripts involved with inflammatory pathways, including neutrophil activation and atherosclerosis signaling. We have adapted a uremic rat model of chronic PD. Chronic PD induced transcriptomic changes related to inflammatory signaling that occur independent of 5/6Nx and augmented circulating triglycerides and predicted atherosclerosis signaling in 5/6Nx LV tissues. The changes are indicative of increased CV risk due to PD and highlight several pathways for potential therapeutic targets.

## Introduction

In the United States more than 780,000 people suffer from end stage kidney disease (ESKD) and each year more than 130,000 people are diagnosed. Of those diagnosed with ESKD, approximately 2.5% receive a kidney transplant, while the remainder are reliant on dialysis for survival^1^. Previous clinical data shows an overall increased survival rate with dialysis; however, there is still a disproportionately high mortality rate compared to the general population. The leading cause of death in the ESKD patient population is cardiovascular disease (CVD)^1^, and over 50% of ESKD deaths are attributed to cardiovascular (CV) related events^2–10^ making the ESKD population one of the largest at-risk groups for CVD and CV related mortality.

There are two main types of dialysis, hemodialysis (HD) and peritoneal dialysis (PD). Approximately 86.5% of new ESKD patients are placed on HD and approximately 11% are placed on PD^1^. Given recent advancements in technology, understanding of peritoneal membrane physiology, and treatments for related complication, PD is gaining support as a more favorable dialysis therapy. It has been suggested that PD should be the primary course of renal replacement therapy, predominantly in the first few years, and in emerging countries or rural populations where access to hemodialysis is limited^9,10^. This shift in dialysis modalities is also reflected in the 2019 executive order on Advancing American Kidney Health which envisions a significant increase in home dialysis utilization for the treatment of ESKD^1^.

Despite advancements, patients receiving PD still have increased CV risk^11–14^. PD patients typically have particularly atherogenic dyslipidemia^15,16^, and current treatments are not effective at reducing the resulting CV risk^17^. CKD has been shown to invoke immune infiltration and alter the innate immune response increasing CV risk^18^. Additionally, many patients on PD experience chronic inflammation and peritonitis, which is understood to result from a combination of dialysate composition, changes in inflammation and the immune system, and infection^19–23^. Several studies utilizing PD in animal models to study these changes have been reported^24–35^, often focused on the impact of dialysate fluid composition on efficacy or peritonitis by repeated, staggered infusion of dialysate into the abdominal cavity. Studies examining the impact of chronic PD, with repeated dwells and withdraws, on CV and renal function are lacking^25,32^. Additionally, there have been no studies looking at molecular changes in heart or kidney tissues in response to chronic PD.

In this study we utilize the 5/6Nx model of CKD to begin to fill these gaps in knowledge with two primary goals: (1) to assess the effects of chronic PD on CV and renal function in animals with and without renal insufficiency and (2) to assess molecular changes in the heart and kidney resulting from chronic PD in both groups. These analyses allow us to better-understand the mechanisms driving increased CV-mortality in ESKD patients receiving PD. This study provides novel evidence that PD increases serum triglycerides while inducing specific transcriptomic changes that associated with downstream inflammation, immune cell infiltration, and atherosclerosis in the left ventricle (LV) all of which are known risk factors for CVD and CV mortality. Our transcriptomic data provide numerous pathways to explore for future therapeutic target development.

## Methods

### Animal model

Animal protocols were approved by the Medical College of Wisconsin Institutional Animal Care and Use Committee (AUA00004133). All work was in adherence to the NIH Guide for the Care and Use of Laboratory Animals and reporting in the manuscript follows the recommendations in the ARRIVE guidelines^36,37^. Male Sprague Dawley rats (N = 6–7/group) (Envigo, Madison, WI) were fed 0.4% NaCl diet (AIN-76A Purified Rodent Diet, Dyets, Inc., Bethlehem, PA) and provided water ad libitum. At 10 weeks of age, rats were anesthetized by i.m. injection of a ketamine (50 mg/kg)/xylazine (8 mg/kg)/acepromazine (5 mg/kg) mixture and subjected to Sham or 5/6Nx surgery using a surgical excision model, as previously described^38,39^. Briefly, a midline incision was made in the abdomen and the right renal artery and vein were ligated with 3.0 silk suture prior to right kidney excision. The left kidney artery and vein were temporarily occluded for scalpel excision of the upper and lower poles of the left kidney. Gelfoam coagulant was applied to the cut surfaces and the occlusion was released. The incision was then closed, and the rat recovered.

### Peritoneal dialysis

**Figure 1:**
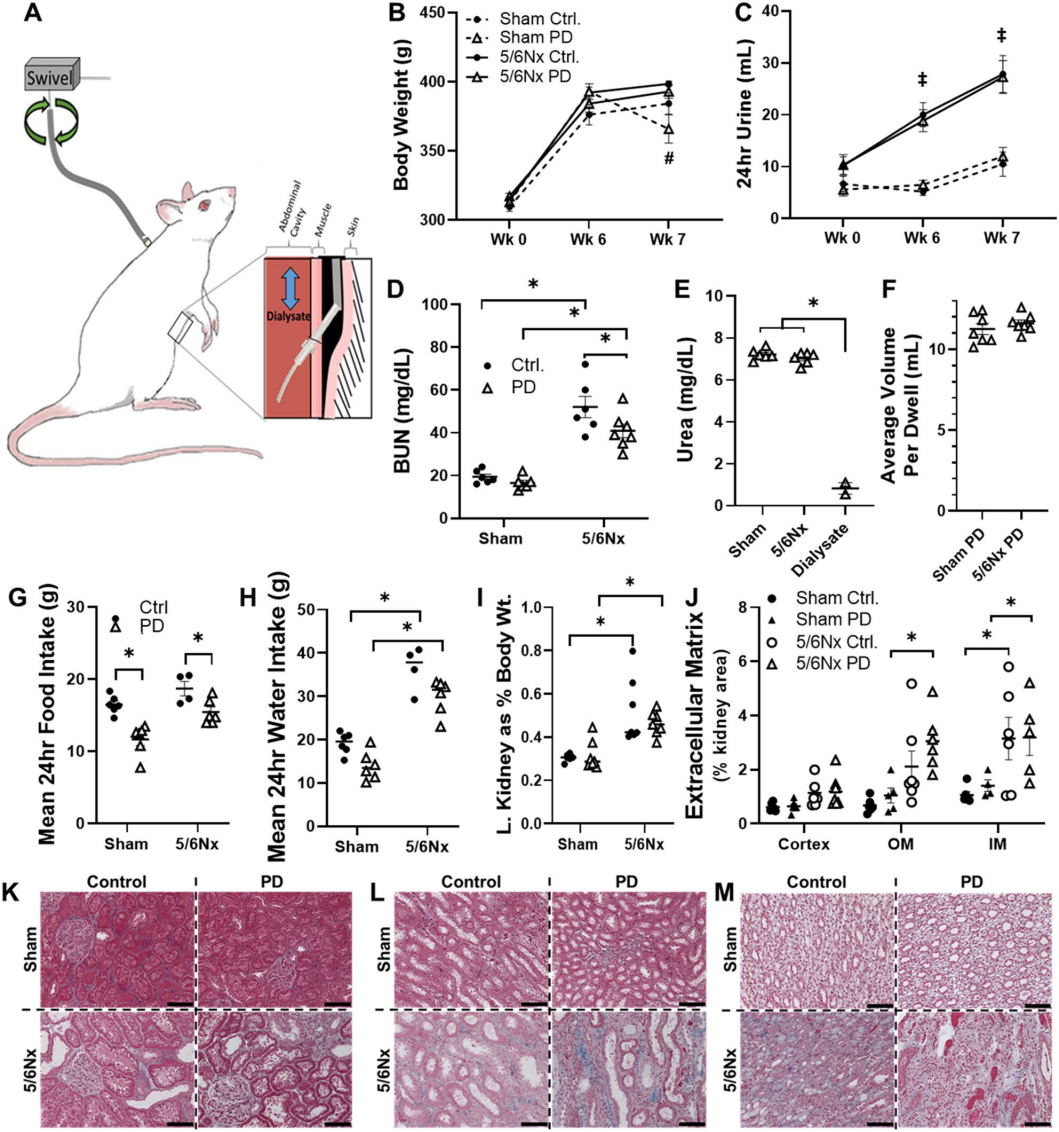
PD efficacy and impact on renal pathology. (**A**) Diagram of PD catheter apparatus and placement. A guide catheter is implanted in the abdominal wall and tunneled subcutaneously to exit between the scapulae. The catheter is then covered by a protective anchored spring attached to a swivel to allow free movement within a metabolic cage for individually housed rats. (**B**) Body weight before surgery (Wk 0), before initiation of dialysis in PD groups (Wk 6) and at the end of the study (Wk 7) and (**C**) matched 24-h urine volumes. (**D**) Reduction of blood urea nitrogen (BUN) and increase in (**E**) urea in recovered dialysate from animals receiving PD indicates that the dialysis strategy was effective in eliminating some metabolic waste. (**F**) The mean volume of recovered dialysate after each 1-h PD dwell within animal. Mean 24-h food intake (**G**) was decreased by PD, regardless of kidney surgery, but PD had no effect on mean 24-h water intake (**H**). (**I**) Body weight normalized (left; L) kidney weight was not impacted by PD in Sham or 5/6Nx groups at the end of the study. (**J**) PD had no effect on renal fibrosis in cortex, outer medulla (OM), and inner medulla (IM) regions of Masson’s trichrome stained kidney sections. Representative images of (**K**) cortex, (**L**) outer medulla (OM), and (**M**) inner medulla. Cal. bar = 100 mm. n = 6–7 rats/group except n = 4–7 (**G**,**H**). Lines represent mean ± SEM. ^‡^*P* < 0.05 5/6Nx vs. Ctrl at indicated timepoints. *P < 0.05 in indicated comparison. Statistical tests include two-way RM ANOVA with Tukey’s post-hoc test (**B**,**C**), two-way ANOVA with Holm–Sidak post hoc test (**D**,**G**,**H**), one-way ANOVA (**E**) or Student’s *t* test (**F**).

At week 6 post-surgery a customized catheter was installed into the abdominal cavity of all Sham or 5/6Nx rats in this study under anesthesia, based on a protocol developed by Motojima et al. (Fig. 1A). All tubing and instrumentation were ethylene oxide sterilized prior to use in surgical procedures or dialysis exchanges. A small midline abdominal incision was made and omentectomy performed. A modified guide tube (Tygon^®^ AJK00004) was inserted into the abdominal cavity through the incision. The guide tube had an ~ 2 mm long exterior tubing section (Tygon^®^ ND-100–65 #ADF00011) glued to the guide tube ~ 3 mm from the bottom to act as an anchor in the abdomen. The guide tube was secured with 3-0 silk suture using a purse string stitch then was exteriorized to the area between scapulae through an implanted wire anchor that was stitched to the back muscle with 3-0 silk suture. A custom PD catheter, the bottom 2/3 inch which was perforated, was then inserted through the guide tube using sterile corn oil as a lubricant. The catheter was covered by a stainless-steel spring for protection and tethered to a swivel apparatus at the top of a metabolic cage, allowing animals to move relatively freely within the cage.

Animals were recovered for 3 days before initiation of PD fluid exchanges with warmed sterile, medical grade dialysate (15 mL; Baxter, PD Dianeal 2, 2.5% dextrose) in one-half of the Sham and 5/6Nx rats (randomly assigned). The other one-half of the animals served as surgical/instrumentation Control but received no dialysate. For those receiving PD, a dialysate exchange was performed 3 times per day for 8 days following catheter implantation. Dialysate was injected into the abdomen slowly through a sterile syringe and needle via the perforated PD catheter. Following a one-hour dwell the dialysis was slowly collected by applying gentle negative pressure with a sterile syringe. After the third exchange each day a new PD catheter was placed in the guide tube. The volume of recovered dialysate was recorded to ensure animals were not retaining significant fluid volume.

### Blood and tissue collection

Animals were euthanized by creating a pneumothorax via thoracotomy under anesthesia (i.m. injection of a ketamine (50 mg/kg)/xylazine (8 mg/kg)/acepromazine (5 mg/kg) and tissues and blood (serum, heparinized plasma, and EDTA-plasma) were collected for downstream analysis. A custom rat chemistry panel was performed by IDEXX (Westbrook, ME) on collected serum. Heparinize plasma was used to measure oxidized low-density lipoprotein (LDL) using an oxLDL ELISA (Mercodia, Winston Salem, NC). Heart and kidney tissue was collected and the wet weight of the (remnant/intact) kidney was recorded. The tissues were divided in half; one half flash frozen in liquid nitrogen for RNA and protein extraction and the other for histology fixed in 10% formalin.

### Histology

Kidneys and ventricle tissue fixed in formalin were sent to the Children’s Research Institute Histology Core at the Medical College of Wisconsin, where they were processed, paraffin embedded, sectioned (4 µM), mounted on slides, and Masson’s trichrome stained. Trichrome stained kidney sections were then imaged at 20 × using an E-400 microscope (Nikon) and SPOT Insight V5.1 digital camera (Diagnostic Instruments). Ventricle sections were scanned using a Super Coolscan 9000 (Nikon). The area of extracellular matrix protein containing (i.e. fibrotic; blue) tissue was quantified using Metamorph software and expressed as fibrosis percentage of total tissue area^40^.

Immunohistochemistry was performed for detection of CD177 antigen, as previously described^38^. CD177 antibody (sc-376329; Santa Cruz Biotechnology) was used at a 1:50 dilution and a rat adsorbed anti-mouse secondary antibody (#BA-2001; Vector Laboratories) was used at a 1:200 dilution. A secondary control slide (no primary antibody; 1:200 secondary antibody) slide was run in parallel with no staining observed.

### Echocardiographic analysis

Echocardiography using a Vivid iq (GE, Waukesha, WI) ultrasound machine was employed to assess LV morphology and function as previously described^38^. LV characteristics (wall thickness, chamber diameter, and ejection fraction) and fractional shortening (FS%) were measured utilizing transthoracic images of the parasternal short-axis view in MM mode.

### Pressure volume analysis

Acute arterial blood pressure and LV pressure–volume analysis was performed using a 2 French ultra-miniature pressure–volume catheter (Millar, Houston, TX) inserted retrograde through the right carotid artery, as previously described^38^. Blood collected in EDTA plasma and warmed to 37 °C was used for volume calibrations after the procedure. Measurements presented as mean of ~ 30 s of stable recording obtained using Labchart PV software (AD Instruments).

### Transcriptomic analysis of LV and kidney tissue

RNA-sequencing was performed by the Genomic Sciences and Precision Medicine Center (GSPMC) and analysis of the sequencing data was performed, as previously described with minor modifications^41,42^. Transcripts with an adjusted p-value (*q*-value) < 0.05 were identified as differentially expressed genes (DEGs) for subsequent pathway analysis, regardless of fold-change. The list of DEGs was entered into Metascape^43^ and Ingenuity Pathway Analysis (IPA) for subsequent analysis. See Supplemental Materials for complete sequencing details and additional information on the Metascape and IPA analysis.

### Statistics

Data are presented as mean ± SEM. analysis was performed with blinding to surgery/treatment status. For each measurement datapoints that were more 2 standard deviations ± the mean of the experimental group it belonged to were excluded from final analysis. Statistical analysis of phenotypic datasets was performed by Student’s *t* test, Two-Way RM ANOVA, Two-Way ANOVA or One-Way ANOVA with Tukey’s multiple comparison or Holm–Sidak post hoc tests, as indicated in Figure Legends, using GraphPad Prism^36^. Differences of *P* < 0.05 were considered statistically significant. Statistical analysis of transcriptomic results was performed as described in Supplemental Materials.

## Results

### PD had no effect on renal function

The effect of PD was evaluated in 5/6Nx rats with renal insufficiency and Sham-operated controls. Indwelling catheters were surgically placed in all animals 6-weeks after the initial surgery, regardless of whether received PD. Bodyweight did not differ between groups prior to Sham or 5/6Nx surgery (week 0) or prior to indwelling catheter placement (week 6). At week 7 body weight tended to be lower (N.S) in Sham PD rats than all other groups (Fig. 1B). While The 5/6Nx surgery increased 24-h urine volume, regardless of PD, there was no difference within Sham or 5/6Nx groups throughout the study (Fig. 1C).

Blood urea nitrogen (BUN), a measure of renal function and PD efficacy, was elevated in the serum in 5/6Nx Control rats compared to Sham Control rats. Chronic PD had no effect on BUN in Sham rats and attenuated the increase in 5/6Nx rats (Fig. 1D). To further assess PD efficacy urea was measured in dialysate recovered from several dwells (Fig. 1E). When compared to fresh dialysate, dialysate recovered from both Sham and 5/6Nx rats contained significantly more urea. Together these data indicate that our PD protocol was effective in clearing some metabolic waste from these animals. Commonly assessed serum analytes values are presented in Supplemental Table 1. Serum creatinine, noted as an imperfect singular indicator of dialysis efficacy^44^, was not significantly reduced by PD in the 5/6Nx rats (Supplemental Table 1).

The volume of recovered dialysate was recorded after each dwell and the average for each animal was plotted (Fig. 1F). Rats whose recovery fell below 10 mL were checked for blockage, and those whose catheters had become blocked were removed from the study. There was no significant difference in the average volume of dialysate recovered.

The average 24-h food and water intake were calculated between weeks 6 and 7 weeks. Sham and 5/6Nx rats receiving PD ate significantly less food than Control rats (Fig. 1G), while water intake was higher in 5/6Nx rats regardless of PD (Fig. 1H).

### Impact of PD on indices of renal pathology

Our previous studies indicated that proximal tubule dilation is a major histological change observed in this 5/6Nx model^38^. Hypertrophic remodeling of the remnant kidney was not impacted by PD, as indicated by body weight normalized left kidney weights (Fig. 1I). Extracellular matrix (ECM) area was evaluated in cortex, inner medulla, and outer medulla to assess the effect of PD on renal pathology, as an index of fibrosis. Renal ECM was increased by 5/6Nx in the inner medulla in PD and Control rats, when compared to corresponding Sham groups, but only in the outer medulla of 5/6Nx rats receiving PD (Fig. 1J). Representative images from cortex (Fig. 1K), outer medulla (Fig. 1L) and inner medulla (Fig. 1M) demonstrate the nature of these differences.

### PD augments serum triglycerides in 5/6Nx rats

**Figure 2:**
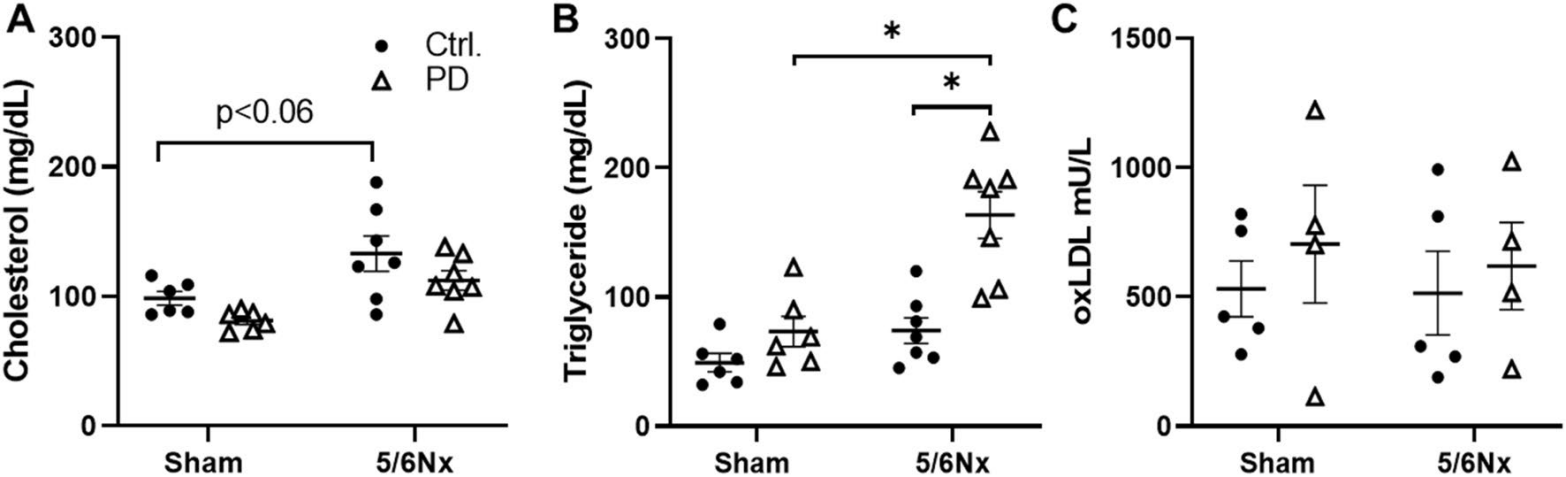
Serum lipid profile 7 weeks post-surgery. (**A**) Serum cholesterol, (**B**) Serum triglycerides, (**C**) Serum oxLDL. N = 4–7 rats/group. Lines represent mean ± SEM. ^‡^*P < 0.05 in indicated comparison. Two-way ANOVA with a Holm–Sidak post hoc test.

Serum cholesterol tended to be higher in 5/6Nx rats, independent of PD (Fig. 2A). By contrast, serum triglyceride levels in 5/6Nx PD rats were nearly two-fold higher than that of Sham Control or 5/6Nx Control rats (Fig. 2B). To further evaluate the lipid profile oxidized LDL was measured from heparinized plasma. There was no difference in oxidized LDL measured between any of the study groups. (Fig. 2C).

### PD did not impact 5/6Nx-associated cardiac remodeling

**Figure 3:**
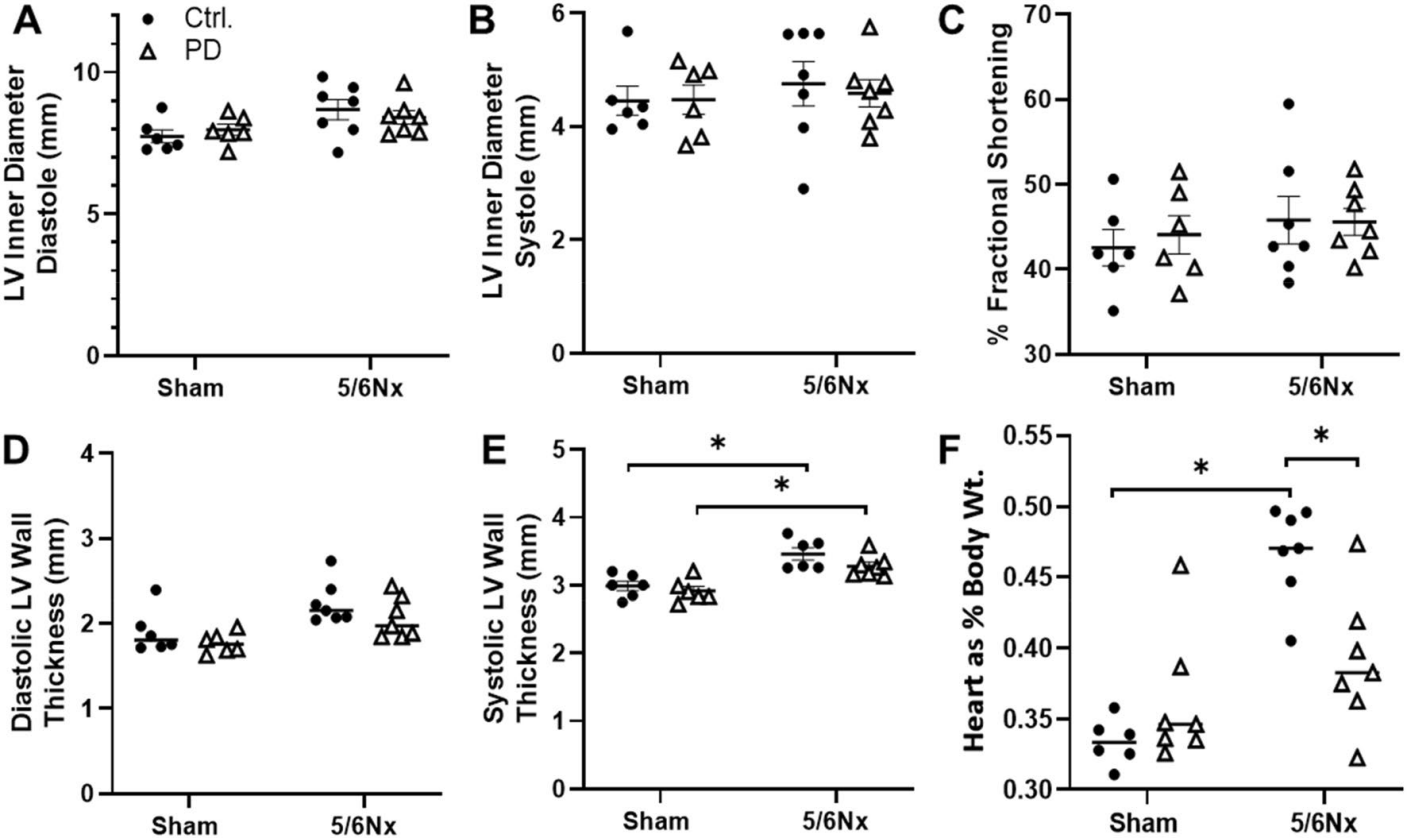
Measures of LV dimensions and function assessed by short-axis echocardiography 7 weeks post-surgery. LV diameter was not altered by PD in (**A**) systole or (**B**) diastole. (**C**) Fractional shortening was also not changed between the groups. Wall thickness (LV) was not different between groups during (**D**) diastole but was significantly higher during (**E**) systole, regardless of PD. The enhanced systolic wall thickness in 5/6Nx Control rats tended to be attenuated by PD (N.S). (**F**) The higher body weight normalized heart weight observed in 5/6Nx Control rats was not observed in 5/6Nx rats receiving PD. PD reduced normalized heart weight. N = 6–7/group. Lines represent mean ± SEM. ^‡^*P < 0.05 in indicated comparison. Two-way ANOVA with a Holm–Sidak post hoc test.

The impact of PD on LV remodeling was assessed in vivo using echocardiography. Diastolic LV chamber diameter was increased in 5/6Nx Control rats compared to Sham rats (Fig. 3A), while there was no difference in systolic chamber diameter (Fig. 3B). Fractional shortening was not altered by either 5/6Nx or PD (Fig. 3C). Diastolic LV wall thickness did not differ between study groups (Fig. 3D), but both 5/6Nx Control and 5/6Nx PD rats exhibited increased systolic LV wall thickness when compared to their respective controls (Fig. 3E). Heart wet weight, as a percentage of total body weight, was higher in 5/6Nx Control rats than Sham rats and 5/6Nx rats receiving PD (Fig. 3F) despite no difference in body weight (Fig. 1B), chamber size (Fig. 3A) or wall thickness (Fig. 3D) between groups.

### PD did not impact blood pressure or LV remodeling, but increased HR only in 5/6Nx rats

**Figure 4:**
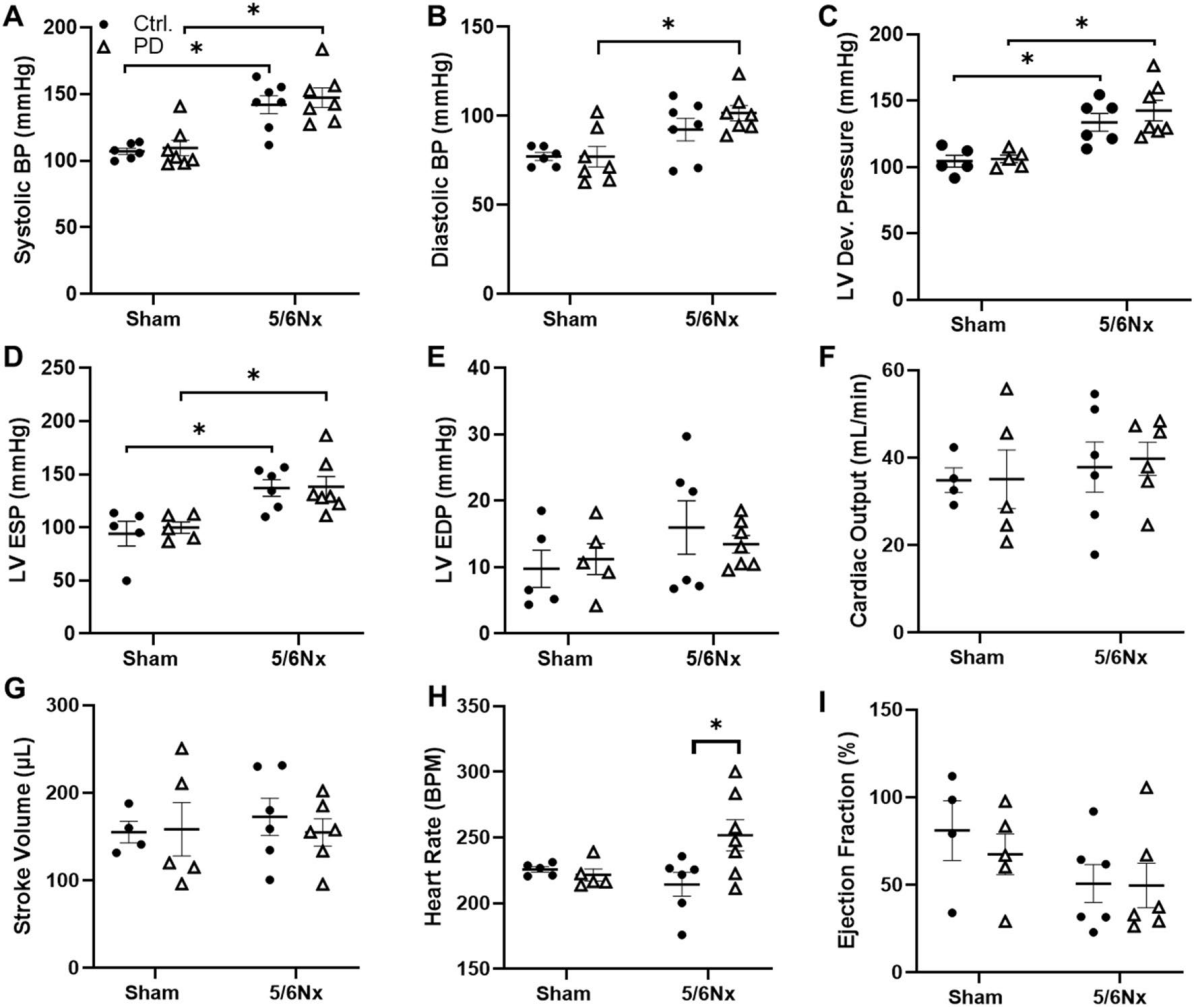
Pressure–volume analysis of the carotid artery and LV 7 weeks post-surgery. Arterial (**A**) systolic blood pressure (BP) and (**B**) diastolic BP was unaffected by PD in Sham and 5/6Nx animals. (**C**) LV developed pressure and (**D**) LV end systolic pressure (ESP) was higher in 5/6Nx animals than Sham but were not impacted by PD. (**E**) LV end diastolic pressure (EDP) did not differ between groups. Neither (**F**) cardiac output nor (**G**) stroke volume was significantly altered by surgery or PD, but (**H**) heart rate was significantly higher in 5/6Nx rats receiving PD. (**I**) Ejection fraction was not different between groups. These data indicate that, at least in this timeframe, the heart is able to compensate for any PD-related hemodynamic shifts. N = 4–7/rats/group. Lines represent mean ± SEM. ^‡^*P < 0.05 in indicated comparison. Two-way ANOVA with a Holm–Sidak post hoc test.

Acute arterial and LV blood pressures (BP) were measured with Millar catheter just prior to endpoint. The catheter was then advanced into the LV through the aorta to measure LV pressures. Systolic BP was elevated in 5/6Nx rats compared to Sham. PD did not affect BP in either Sham or 5/6Nx rats (Fig. 4A). By contrast, diastolic arterial BP was only elevated in 5/6Nx PD rats compared to Sham PD rats (Fig. 4B).

Developed LV pressure was elevated in 5/6Nx rats compared to Sham rats, with or without PD (Fig. 4C). This was due to increased LV end systolic pressure (Fig. 4D), as end diastolic pressure was unchanged (Fig. 4E). End systolic LV pressure was elevated in 5/6Nx rats compared to Sham. This moderate increase in afterload did not impact cardiac output (Fig. 4F), or stroke volume (Fig. 4G); however, PD significantly increased heart rate in 5/6Nx rats (Fig. 4H). Ejection fraction was not altered by PD in either surgical group (Fig. 4I).

### Transcriptomic analysis of LV and kidney tissues in sham vs. 5/6Nx rats with and without PD

RNA sequencing yielded > 50 million reads (100 nucleotides in length) per sample, with a sequencing depth sufficient to accurately quantify most transcripts and variants^45^. Sequencing data files and processed files are available in GEO (GSE207524). The mapping rate for this dataset was > 98% for all samples (Supplemental Table 2). The number of transcripts with an adjusted *P*-value < 0.05 in each comparison (Differentially Expressed Genes; DEGs) are indicated in Supplemental Table 3, along with the number of mapped transcripts in pathway analysis. Changes induced by PD in the Sham-operated animals resulted in the largest number of DEGs in both LV and kidney tissues (853 and 637, respectively). By contrast 162 LV and 33 kidney DEGs were identified in 5/6Nx PD versus 5/6Nx Control comparisons.

**Figure 5:**
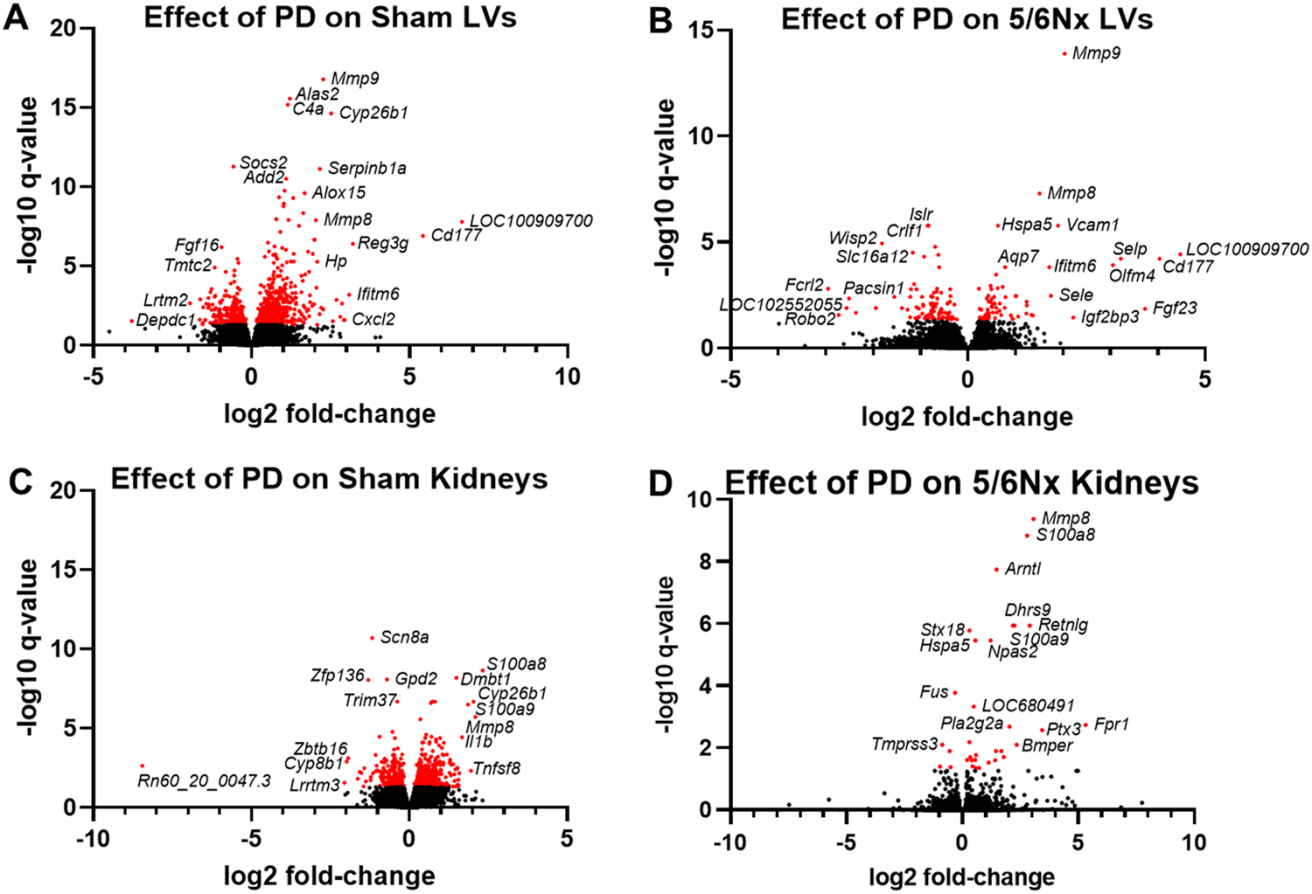
Alterations in transcript expression in rats receiving PD compared to those that did not in (**A**) Sham LV, (**B**) 5/6Nx LV, (**C**) Sham kidney and (**D**) 5/6Nx kidney tissues. In these comparisons − log10 *q*-value were plotted against log2 fold-change of PD relative to no PD comparison, indicating significance of difference and fold-change of difference, respectively. N = 6–7/ rats/group. Points representing DEGs (*q* < 0.05 PD vs. Control) are indicated in red.

The log2 fold-change of the difference in transcript expression in PD versus Control comparisons was plotted against − log10 q-value (significance of difference) for Sham LV (Fig. 5A), 5/6Nx LV (Fig. 5B), Sham Kidneys (Fig. 5C) and 5/6Nx Kidneys (Fig. 5D). Those transcripts that were significantly altered (*q* < 0.05) are indicted in red. DEGs with the top 15 largest log2 fold-change, in each comparison are listed in Supplemental Tables 4, 5, 6 and 7, with corresponding *P*-value and FDR-adjusted p-value.

The DEGs identified from each comparison were analyzed by IPA and Metascape pathway tools. IPA identified the predicted activation of numerous known relationships between upstream regulators, diseases, functions, and pathways identified during analysis based on the directionality of transcript expression changes. IPA results of canonical pathway enrichment was coded by positive (orange) or negative (blue) z-score (i.e., activation score). Metascape analysis identified numerous enrichment terms based upon the DEG input.

A graphical summary generated by IPA (A) is followed by a list of enriched functional terms generated by Metascape (B) for Sham LV (Supplemental Fig. 1), 5/6Nx LV (Supplemental Fig. 2), Sham Kidney (Supplemental Fig. 3), and 5/6Nx Kidney (Supplemental Fig. 4).

**Figure 6:**
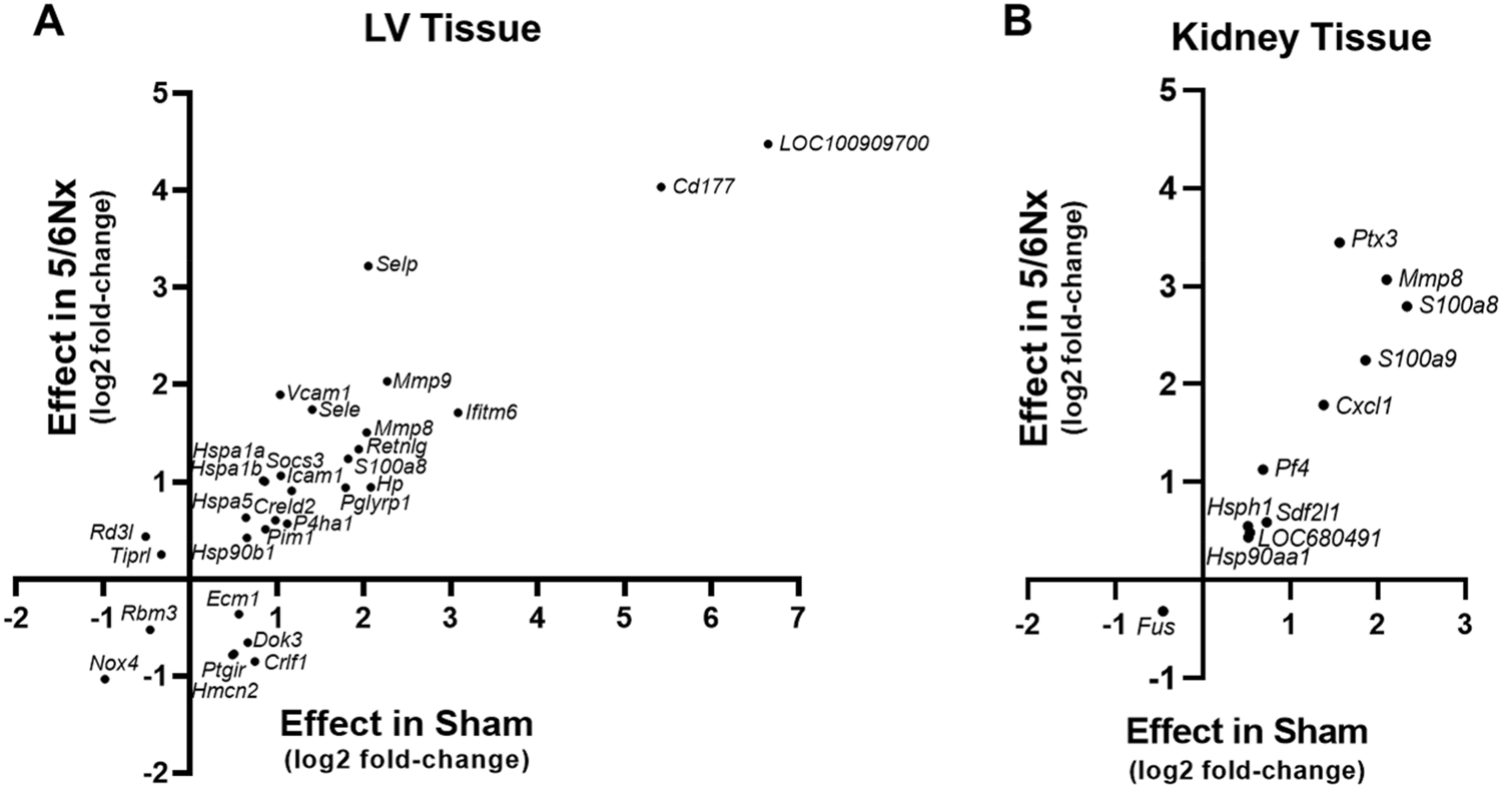
DEGs altered in both Sham and 5/6Nx rats in response to PD. (**A**) LV tissues shared more DEGs than (**B**) kidney tissues. N = 6–7/rats/group. Points representing log2 fold change in each tissue type.

To identify those alterations that were preserved in both Sham and 5/6Nx rats and unique to each tissue, we plotted log2 fold-change of DEGs common to both 5/6Nx and Sham rats in either the LV (Fig. 6A) or kidney (Fig. 6B). This analysis identified a large upregulation of LV *Cd177* and *LOC100909700* transcripts by PD in either Sham or 5/6Nx rats.

**Figure 7:**
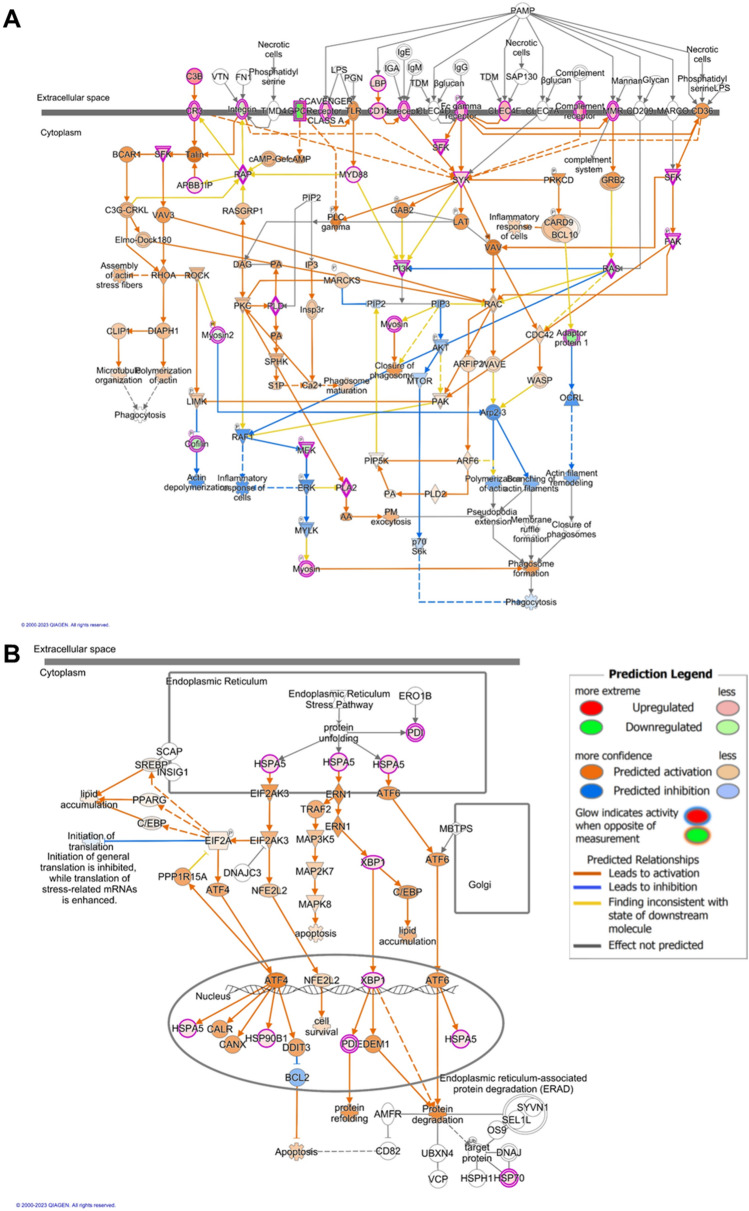
Pathways with highest predicted activation in LV tissue, as determined by Ingenuity Pathway Analysis (IPA; Qiagen). (**A**) “Phagosome Formation” had the highest activation z-score in PD vs. Ctrl. comparisons in Sham rats, while (**B**) “Unfolded Protein Response” had the highest activation z-score in PD vs. Ctrl. comparisons in 5/6Nx rat.

The IPA tool was also used to examine documented pathways that were enriched in differentially expressed genes. Figure 7 depicts the pathways that had the highest activation z-score in LV comparisons, meaning they had the highest predicted activation with PD, relative to no PD samples. In LV tissue from sham rats, the “Phagosome Formation” pathway (Fig. 7A) was predicted to be activated by PD (z-score of 4.082, p-value of 4.16E-09, and representing 54/691 genes). In LV tissue from 5/6Nx rats, the “Unfolded Protein Response” pathway (Fig. 7B) was predicted to be activated by PD (z-score of 2.236, p-value of 5.49E-04, and representing 5/77 pathway genes).

**Figure 8:**
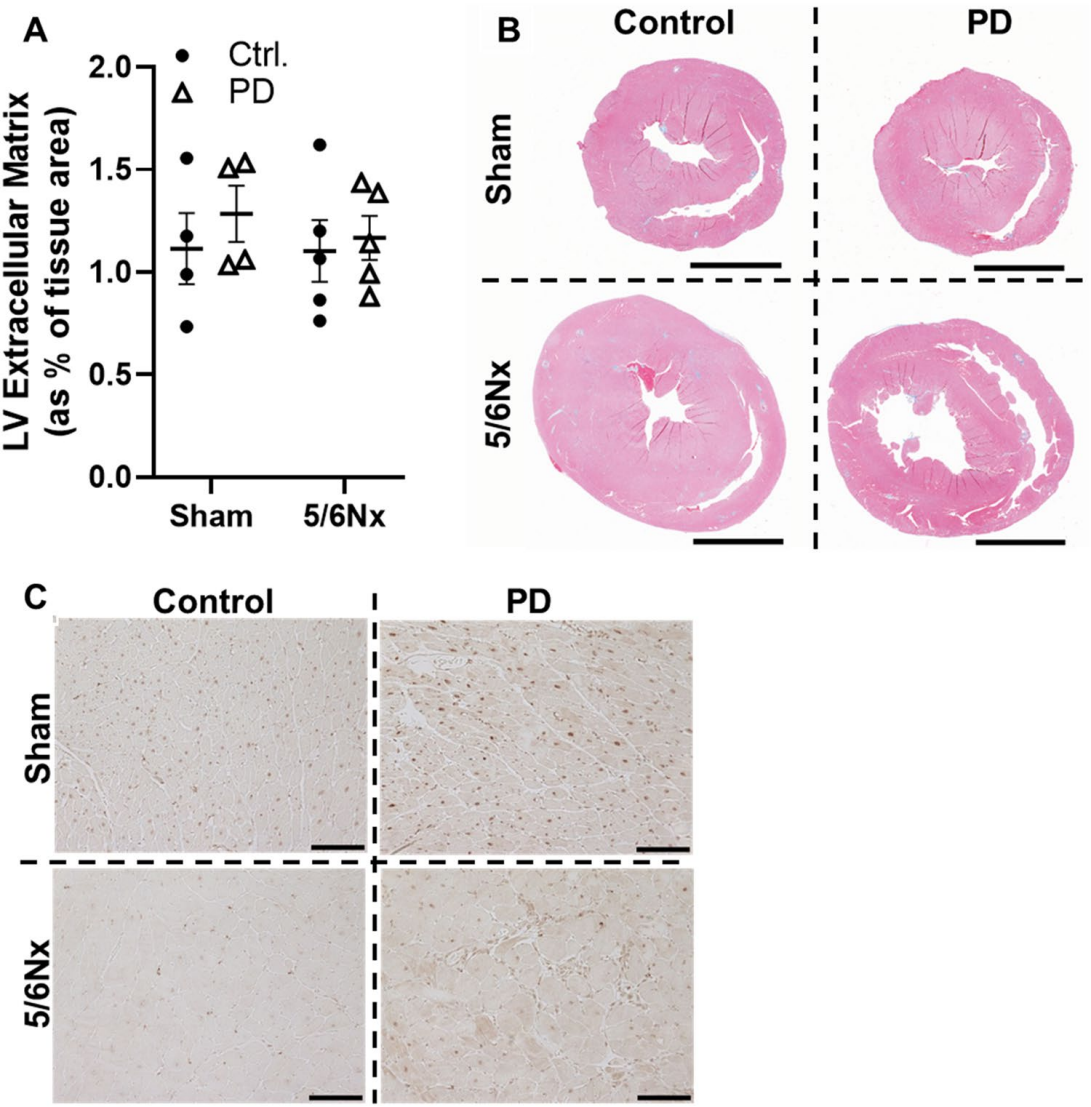
Alterations in Sham and 5/6Nx LV tissue in response to PD. (**A**) The percent of LV tissue exhibiting fibrosis with Masson’s trichrome staining was not altered by 5/6Nx and/or PD. (**B**) Representative scans of trichrome stained ventricle cross-sections. Cal. bar = 5 mm. (**C**) Immunohistochemistry for CD177 antigen detection in LV confirms transcriptomic profiling at the protein level. Staining was observed throughout the myocardium and was upregulated by PD in LV tissue from both Sham and 5/6Nx. Positive CD177 antigen signal was enriched within cardiomyocyte nuclei in Control animals. PD increases nuclear and cytoplasmic expression in LV tissue from Sham rats, while primarily increasing cytoplasmic staining in LV tissue from 5/6Nx rats. Cal. bar = 100 mm. N = 4–5/group. Lines represent mean ± SEM. ^‡^*P < 0.05 in indicated comparison. Two-way ANOVA with a Holm–Sidak post hoc test.

There was no indication of LV fibrosis in any of our experimental groups (Fig. 8A), nor apparent differences in regional distribution of extracellular matrix proteins (Fig. 8B). Immunohistochemistry of LV tissue revealed that CD177 antigen expression was augmented by PD regardless of Sham or 5/6Nx surgery (Fig. 8C). We observed that staining was weak and largely localized to cardiomyocyte nuclei in Control tissues; nuclear signal was higher in the Sham group (left images, Fig. 8C). The LV tissues from Sham rats receiving PD showed augmented CD177 signal, primarily in the nucleus. The LV tissues from 5/6Nx rats receiving PD also had augmented CD177 signal, but the nuclear signal was weaker than in the Sham PD group and distributed primarily through the cytoplasm of the cardiomyocytes.

## Discussion

Currently only 11% of the dialysis population receives PD for renal replacement therapy^1^, however it has become more favorable with recent advancements^9,10^. Additional studies are needed to better understand the systemic and multi-organ effects of this dialysis modality. Chronic inflammation is common in patients receiving PD, even in those without peritonitis^46^. Over time this can lead to thickened PD membranes and reduced diffusion efficiency^47,48^. Inflammation has been highly associated with CV events^49^ and mortality^50^ in PD patients.

In this study we utilized the 5/6Nx model of renal insufficiency and CKD to explore the CV and renal effects of PD, hypothesizing that chronic PD would worsen CV parameters in 5/6Nx rats. Because inflammation is a common complication in PD patients^46^, we implanted a catheter in Control rats though they did not receive dialysate to account for surgery-related inflammation. The results of our study provide several novel and important advancements to the field of PD research. First, this is one of only a few reported studies^25,32^ where chronic PD with exchanges was performed in a rodent model. This approach can be adopted by other researchers to study PD complications and optimize therapies. Second, this is the first study assessing the effect of chronic PD with exchanges on CV function. Finally, this is the first reported study assessing the effect of chronic PD on renal and LV pathology and transcriptomes.

Analysis of indices of renal function suggests that, while PD provides some toxin clearance, it does not improve or prevent renal damage. As with an earlier study in this model by our group, 5/6Nx caused LV hypertrophy^38^. The sum of our LV analysis found no effect of 5/6Nx or PD on cardiac function, indicating that animals were able to compensate for PD-related changes in hemodynamic load. An attenuation of hypertrophic remodeling was observed, but it is not clear from this study if that will have an impact on functional outcomes in the longer term. We did observe that PD increased heart rate in 5/6Nx rats, without a change in stroke volume, ejection fraction or cardiac output. This may be an indication that PD animals are experiencing, and compensating for, CV stress.

Elevated blood lipids have been associated with increased CV risk in CKD patients^51^. In this study we found that PD increased serum triglycerides in 5/6Nx, but not Sham rats. This is an important finding because ESKD patients receiving PD often present with hyperlipidemia with increased triglycerides, and low HDL levels. In addition, VLDL, IDL, LDL and total cholesterol can be elevated, but more importantly the LDL particles are typically small, dense, and easily oxidized, making for a highly atherogenic lipid profile^15,16,52^. While current literature is aware of the unique lipid profile that develops in the PD population it is not known how PD may directly influence the changes. Current belief is that glucose absorption from the dialysate may result in lipoprotein synthesis^15,16^, however, our data suggest this may not be the primary mechanism as triglycerides are significantly increased in the 5/6Nx rat with a reduction in serum glucose. It is clear in this model that an interaction between renal insufficiency and PD result in large triglyceride increases. Studies exploring the impact and mechanism of the increased triglycerides utilizing this model could clarify their possible role in worsening CVD in this population and help to identify new therapeutic strategies. Current lipid treatment recommendations from KDIGO for PD patients suggest that statin treatments are not effective at reducing CV risk and while triglyceride targeting medications like fibrates can reduce risk the increase in plasma creatinine contraindicates them for use in this population^17^. Therefore it is imperative to better understand the mechanisms involved in the development of the unique lipid profile for the development of improved therapeutics.

We noted a reduction in food intake in animals receiving PD, though only Sham-operated PD rats tended to experience some weight loss. It had been previously shown in a rat model of peritoneal infusion of dialysate that large dialysate volumes (30 mL) and hyperosmolarity were not responsible for reduced ingestion behaviors, rather glucose absorption from dialysis may importantly contribute to reduced appetite^53^. Clinical studies have noted that patients receiving PD absorb glucose from the dialysate^54^, which may reduce appetite^55^ and complicate glucose management in diabetic patients^56,57^. With all of that considered, the reason for the reduced body weight in Sham PD rats in this study is unknown and serum glucose was not elevated following PD in our study.

The transcriptomic analysis completed in this study provided novel insight into organ-specific molecular changes that occur in response to PD. Pathway analysis predicted activation of inflammatory and immune pathways by PD regardless of tissue type or renal status. One of the most notable aspects of this study was the substantial impact of PD on LV transcript expression in Sham rats despite the lack of measurable changes in CV or renal parameters.

Our data suggests that chronic delivery of dialysate induced numerous changes in gene expression unique to sham or 5/6Nx groups, but also common to both surgical groups. The PD-induced transcriptomic changes specific to either the LV or kidney tissues, but common to Sham and 5/6Nx rats were analyzed in greater detail. When we compared either LV or kidney DEGs resulting from PD in Sham-operated rats against 5/6Nx rats we found a subset of transcripts that were clearly impacted by PD. In LV tissues, regardless of surgery, PD increased several heat shock protein, selectins, and matrix metalloproteinase transcripts, among others, and decreased *Rbm3* and *Nox4* transcripts. The transcripts that were most highly upregulated by PD in the LV in both surgical groups were *Cd177* and *LOC100909700* (also provisionally renamed *Cd177* in the Rat Genome Database^58^). This was not observed in kidney tissues. The CD177 antigen has been reported as a specific marker of neutrophil activation in studies focused on leukocyte markers^59,60^; however, we observed expression throughout the LV myocardium. CD177 antigen has been shown to be expressed and functionally important in other cell types^61,62^. Immunohistochemistry images shared in the Human Protein Atlas^63^ reveals faint nuclear staining in cardiac tissues, similar to that of our Control rat LV tissues. While its function is not well understood^64^, particularly in non-neutrophil cell types, CD177 has been reported to regulate cell mammary epithelial proliferation^61^ and is known to bind several proteins, notably platelet endothelial cell adhesion molecule 1 (PECAM-1, also known as CD31)^65^.

Neutrophils also play an important role in the development of atherosclerosis^66^. Neutrophil rolling is mediated by endothelial selectins (*Sele* and *Selp*) and integrins^67^, which were also upregulated by PD. Pathway analysis (IPA) identified “Atherosclerosis Signaling” pathway as the most highly enriched in DEGs in 5/6Nx rats.

It is also possible that these changes highlight neutrophil dysfunction, which have been observed in individuals receiving chronic dialysis^68^. Uremic toxins^69^ and dialysate^70^ can both impair neutrophil function. We also observed that fibroblast growth factor 23 (*Fgf23*) transcript was significantly increased by PD in LV tissue from 5/6Nx, but not Sham rats. FGF23 helps to maintain phosphate homeostasis during CKD^71^ and induce pressure-independent cardiac hypertrophy^72^. Elevated FGF23 has also been associated with infections and mortality in hemodialysis patients^73–75^, possibly through binding to surface receptor FGFR2^76^. Further studies will be needed to understand the impact of these changes in gene expression on CV pathology and neutrophil function in CKD in the presence and absence of PD in this model. These data provide an array of pathways to explore in the future consideration of therapeutic targets when considering inflammation, immune infiltration, and atherosclerosis as risk factors for CVD and mortality in the PD population.

IPA analysis was used to identify pathways with a high activation z-score in PD versus no-PD comparisons, “Phagosome Formation” and “Unfolded Protein Response” pathways were predicted to be activated in LV tissue from Sham and 5/6Nx rats, respectively. A recent study of skeletal muscle phenotypes in patients receiving chronic dialysis noted the presence of autophagosomes^77^. This may suggest that activation of phagosome formation would be detrimental to cardiac muscle, though autophagy has been primarily reported protective against peritoneal fibrosis^78–81^, a common complication of chronic PD therapy. A role for activated “Unfolded Protein Response” in response to cardiovascular stress is well-established and has been reviewed extensively^82,83^. In this study it is notable that chronic PD is augmenting this response in the 5/6Nx rats, which already exhibit substantial cardiac pathology. These are just two examples of disease-related mechanisms that have been identified through transcript profiling from data generated in this study.

The 5/6Nx model differs substantially from ESKD that may develop over years. Despite this limitation, 5/6Nx provides the flexibility to initiate PD during progression renal pathology (6 weeks post-surgery) rather than abruptly initiating PD following total nephrectomy which could introduce substantial acute hemodynamic stress. Given the robust changes in gene expression observed with PD, extension of the dialysis period beyond week 7 of the study may reveal alterations in CV function or pathology attributable to PD. The 8-day window of PD was selected because it followed the development of renal insufficiency and spanned a period of pathological LV changes^38^. In our study PD reduced heart weight/body weight and increased heart rate in the 5/6Nx group, but did not alter other measures including LV wall thickness, chamber diameter, pressure, or ejections. Our results suggest that an extended duration of chronic PD may be needed to observe a functional impact of chronic PD-related inflammation on LV remodeling and function.

The development and optimization of this model was completed in adult male rats; thus, this study cannot provide insight on the effect of HD in adult female rats. It will be important to understand any sexual dimorphism in pathophysiological response to PD, since female sex hormones have been reported to be cardio- and reno-protective^84–86^ and augment immune response^87–89^. Optimization studies in female rats will be needed to appropriately modify instrumentation and dwell volumes for smaller animals, and to prevent catheter blockage that may result from peri-ovarian adipose.

In summary, these studies highlight the importance of a continued exploration into the effects of dialysis and identifying therapeutic targets to improve CV outcomes in the ESKD population. We find that chronic PD increases serum triglycerides in the 5/6Nx rat altering the lipid profile similar to that seen in the clinical population. Additionally, PD alters transcript expression in the LV and kidney, even in the absence of renal insufficiency. Furthermore, PD leads to gene changes suggesting an enhanced inflammatory response, activated immune infiltration, and atherosclerotic signaling, all of which are linked to increased CV-mortality risk. While these changes did not compromise cardiac function over the duration of this study, they may contribute to added CVD and cardiac pathology and mortality over an extended period. Exploration of these pathways and a better understanding of the mechanisms through which they are altered can lead to the development of potential therapeutics to ultimately reduce CV risk and mortality in this population.

## Supplementary Information

Supplementary Information 1.

Supplementary Information 2.

## Data Availability

Raw data points collected for phenotypic analysis are provided in the Supplementary Raw Data file. Transcriptomic datasets generated and/or analyzed during the current study are available in the. Gene Expression Omnibus (GEO; Accession #GSE207524) and (SRA; Accession #PRJNA855957).
