# Supplementary material for: Transcriptomic analysis identifies novel candidates in cardiorenal pathology mediated by chronic peritoneal dialysis
Source: Sci Rep. 2023 Jun 21;13:10051. doi: 10.1038/s41598-023-36647-7 (PMC10284882; doi:10.1038/s41598-023-36647-7)
Supplement: Supplementary file 2 — Supplementary Information 2. [file 41598_2023_36647_MOESM2_ESM.pdf]

**SUPPLEMENTAL MATERIAL**

# Transcriptomic Analysis Identifies Novel Candidates in Cardiorenal Pathology Mediated by Chronic Peritoneal Dialysis

Victoria L. Nasci[1,2], Pengyuan Liu[1], Amanda M. Marks[1], Adaysha C. Williams[1], Alison J. Kriegel[1,3,4,5]

[1] Department of Physiology, Medical College of Wisconsin, Milwaukee, WI

[2] Division of Nephrology and Hypertension, Vanderbilt University Medical Center, Nashville, TN.

[3] Cardiovascular Center, Medical College of Wisconsin, Milwaukee, WI

[4] Department of Pediatrics, Medical College of Wisconsin, Milwaukee, WI

[5] Center of Systems Molecular Medicine, Medical College of Wisconsin, Milwaukee, WI

Supplemental Material Table of Contents:

Supplemental Methods

Supplemental Data

Tables S1-S4

Figures S1-S4

Supplemental References

**Supplemental Methods:**

*Additional Details of RNA-sequencing*

Frozen tissues were pulverized under liquid nitrogen, with ~100mg placed in Trizol® for RNA extraction following manufacturer's instructions (Ambion™). Samples were submitted to Genomic Sciences and Precision Medicine Center (GSPMC) for RNA quality assessment using an RNA Fragment Analyzer and quantification by Qubit (ThermoFisher Scientific). A total of 500 ng of high-quality RNA from each sample was used for automated mRNA library construction utilizing the Illumina TruSeq stranded mRNA library prep kit with unique dual 8 base pair indexes for samples on each run. The library quality was evaluated by Kapa qPCR Quantification, MiSeq Nano 50 cycle QC run, prior to proceeding with sequencing. Samples were multiplexed 64 samples per flow cell and pair-end sequenced with an Illumina NovaSeq6000 sequencer.

In analysis, adapter sequences were removed from the output reads; sequences with low quality (base quality < 13) at both ends of reads were further trimmed and trimmed reads with less than 25 bp were removed using the Trim Glore tool [1]. The trimmed short sequencing reads were aligned to the rat reference (rn6) using HISAT2 (v2.2.1) with mammalian default parameters [2]. Transcript construction, quantification and normalization of transcript abundance was performed using StringTie2 (v2.1.5) [3].

Statistical analysis of sequencing results was performed comparing LV RNA from 5/6Nx rats that received PD and those that did not. This comparison was also made in remnant kidney RNA from these groups. Differential gene expression was determined using the R package DESeq2 [4]. The Benjamini-Hochberg method was used to control false discovery rate in differential expression analysis to provide an adjusted *P*-value.

*Ingenuity Pathway Analysis*

A list of DEGs with corresponding log2 fold-change for each comparison of interest was uploaded to IPA for core analysis utilizing the “Ingenuity Knowledge Base” (IPA; QIAGEN, Hilden, Germany) as the “Reference Set”. Data resulting from IPA Graphical Summary in the Subcellular view are presented here, which allows visualization of enrichment established pathways with DEGs from a given data set. The enrichment is ranked within these pathways to provide a “-log (*P*-value)” for enrichment based on the # of differentially expressed genes in the dataset relative to the overall number of genes represented in the pathway. Specifically, in this analysis, we utilized the “Graphical Summary” graphics with default settings, which include a color-coding of pathways that are predicted to be activated or inhibited based on “z-score”. This z-score is also referred to as the activation score because it is used as an index of biological function based on established directionality of effects resulting from gene-gene interactions.

*Metascape Analysis*

The list of DEGs from each comparison group were uploaded and species R. norvegicus was selected for both input and analysis. Express analysis was then performed. The Metascape [5] tool then utilized this input information to generate “Enriched Ontology Clusters” for graphical presentation of datasets.

## Supplemental Data

*Sequencing Results*

The mapping rate for this dataset was >96% for all samples (Table S1). The number of genes with an adjusted *P*-value <0.05 in each comparison (Differentially Expressed Genes; DEGs) are indicated in Table S2, along with the number of mapped genes from this list that represented in Metascape, and IPA tools used in pathway analysis.

*Changes in LV Tissue resulting from PD in Sham and 5/6Nx Rats.*

The LV DEGs with the top 15 largest log2 fold-change increase or decrease in response to PD in either the Sham group or the 5/6Nx group are listed in Tables S3 and S4, respectively. Also listed are the *P*-value and adjusted *P*-value for that comparison. The IPA Graphical Summary Representation highlights numerous processes and pathways are predicted to be activated (orange) or inhibited (blue) with PD based DEGs in Sham (Figure S1A) and 5/6Nx rats (Figure S2A). Metascape analysis identified numerous enrichment terms based upon the same DEG input from our LV tissues in Sham (Figure S1B) and 5/6Nx rats (Figure S2B).

*Changes in Kidney Tissue Resulting from PD in Sham and 5/6Nx Rats.*

We performed the same analysis with the DEGs from kidney tissues as described above for LV tissue. This Metascape analysis yielded fewer enriched pathways and there were not clear pathological processes identified in both Sham (Figure S3A) and 5/6Nx rats (Figure S4A). The enrichment through IPA analysis of Sham kidneys almost

exclusively represented upregulated genes (Figure S3B). When MAP was applied to the 5/6Nx kidney dataset there were no pathways that were predicted to either be activated or inhibited based on our limited input (Figure S4B).

**Table S1**. Serum analyte concentrations at the end of the study.
*$P$ <0.05 vs. 5/6Nx Control, $^{\#}P$ <0.05 vs. Sham Control, $^{‡}P$ < 0.05 vs. Sham PD, Two-way ANOVA

| **Serum Value** (concentration) | **Sham Control** (*n*=6; Mean ± SEM) | **Sham PD** (*n*=6; Mean ± SEM) | **5/6Nx Control** (*n*=7; Mean ± SEM) | **5/6Nx PD** (*n*=7; Mean ± SEM) |
|---|---|---|---|---|
| Alkaline Phosphatase (U/L) | 96.50 ± 8.21 | 129.67 ± 11.14 | 146.29 ± 9.74 | 145.71 ± 19.05 |
| Aspartate Aminotransferase (U/L) | 119.00 ± 10.41 | 128.67 ± 15.58 | 103.57 ± 13.53 | 108.43 ± 9.68 |
| Alanine Aminotransferase (U/L) | 24.83 ± 1.01 | 22.50 ± 3.60 | 17.43 ± 2.03 | 21.14 ± 1.70 |
| Biliruben (mg/dL) | 0.15 ± 0.03 | 0.18 ± 0.02 | 0.11 ± 0.01 | 0.13 ± 0.02 |
| Creatinine (mg/dL) | 0.37 ± 0.02 | 0.41 ± 0.03 | 1.16 ± 0.19$^{\#}$ | 0.84 ± 0.05$^{‡}$ |
| Albumin (g/dL) | 2.42 ± 0.03 | 2.18 ± 0.07 | 2.19 ± 0.08 | 2.17 ± 0.05 |
| Total Protein (g/dL) | 5.12 ± 0.05 | 4.72 ± 0.11 | 4.84 ± 0.13 | 4.73 ± 0.07 |
| Globulin (g/dL) | 2.70 ± 0.06 | 2.53 ± 0.07 | 2.66 ± 0.08 | 2.56 ± 0.04 |
| Glucose (mg/dL) | 248.83 ± 4.90 | 234.00 ± 20.01 | 266.43 ± 6.80 | 204.14 ± 11.54* |
| Calcium (mg/dL) | 9.00 ± 0.06 | 9.02 ± 0.15 | 9.49 ± 0.20 | 9.16 ± 0.09 |
| Phosphorous (mg/dL) | 8.45 ± 0.08 | 10.50 ± 0.26 | 11.13 ± 1.16$^{\#}$ | 10.13 ± 0.30 |
| Bicarbonate (mmol/L) | 24.17 ± 0.31 | 26.83 ± 2.12 | 24.86 ± 1.30 | 26.43 ± 2.08 |
| Chloride (mmol/L) | 101.67 ± 0.84 | 102.50 ± 0.76 | 106.00 ± 1.23$^{\#}$ | 103.43 ± 0.53 |
| Potassium (mmol/L) | 5.60 ± 0.15 | 6.43 ± 0.19$^{\#}$ | 5.86 ± 0.26 | 6.44 ± 0.16$^{\#}$ |
| Sodium (mmol/L) | 145.67 ± 0.80 | 146.00 ± 0.52 | 145.43 ± 0.43 | 144.71 ± 1.13 |

**Table S2.** Summary of RNA-Sequencing Results

| Tissue | Treatment | Sample ID | Yield (Gb) | # Reads | % of >= Q30 Bases | Mean Quality Score | mapping rate (%) |
|---|---|---|---|---|---|---|---|
| LV | 5/6Nx | 1 | 5.9 | 58,670,556 | 99.2 | 36.1 | 97.3 |
| LV | 5/6Nx | 2 | 6.1 | 61,034,214 | 99.3 | 36 | 97.6 |
| LV | 5/6Nx | 3 | 6.9 | 69,057,502 | 99.2 | 36 | 97 |
| LV | 5/6Nx | 4 | 7.7 | 77,016,750 | 99.3 | 36 | 97.6 |
| LV | 5/6Nx | 5 | 11.6 | 116,751,842 | 99.2 | 36 | 97.2 |
| LV | 5/6Nx | 6 | 5.6 | 55,906,430 | 99 | 36 | 97.1 |
| LV | 5/6Nx | 7 | 5.6 | 56,082,226 | 99.2 | 36 | 97.5 |
| LV | 5/6Nx PD | 8 | 7.5 | 75,232,624 | 99.3 | 36 | 97.6 |
| LV | 5/6Nx PD | 9 | 6.2 | 61,581,034 | 99.2 | 36 | 97.5 |
| LV | 5/6Nx PD | 10 | 5.2 | 52,258,372 | 99.3 | 36 | 97.6 |
| LV | 5/6Nx PD | 11 | 5.9 | 59,612,720 | 99.2 | 36 | 97.2 |
| LV | 5/6Nx PD | 12 | 7.3 | 72,620,704 | 99.1 | 36 | 97.3 |
| LV | 5/6Nx PD | 13 | 5.2 | 52,407,500 | 98.9 | 36 | 96.9 |
| LV | 5/6Nx PD | 14 | 6.5 | 65,177,972 | 98.9 | 35.8 | 96.9 |
| Kidney | 5/6Nx | 1 | 7 | 69,769,332 | 99.1 | 36 | 97.8 |
| Kidney | 5/6Nx | 2 | 6.9 | 69,113,072 | 99.4 | 36.1 | 97.9 |
| Kidney | 5/6Nx | 3 | 6.6 | 66,424,826 | 99.4 | 36.1 | 97.6 |
| Kidney | 5/6Nx | 4 | 6.2 | 62,048,520 | 99.4 | 36.1 | 97.9 |
| Kidney | 5/6Nx | 5 | 6.3 | 63,117,042 | 99.1 | 36 | 97.4 |
| Kidney | 5/6Nx | 6 | 6.3 | 63,497,384 | 99.1 | 36 | 97.6 |
| Kidney | 5/6Nx | 7 | 6.1 | 61,021,826 | 99.3 | 36 | 98.1 |
| Kidney | 5/6Nx PD | 8 | 7.1 | 71,013,656 | 99.4 | 36 | 98.1 |
| Kidney | 5/6Nx PD | 9 | 6.5 | 65,368,892 | 99.1 | 36 | 97.7 |
| Kidney | 5/6Nx PD | 10 | 6.3 | 62,776,408 | 99.3 | 36 | 97.8 |
| Kidney | 5/6Nx PD | 11 | 5.4 | 54,316,070 | 99.3 | 36 | 97.9 |
| Kidney | 5/6Nx PD | 12 | 7.9 | 79,041,266 | 99.2 | 36.1 | 97.5 |
| Kidney | 5/6Nx PD | 13 | 6.7 | 67,136,454 | 99 | 36 | 97.4 |
| Kidney | 5/6Nx PD | 14 | 6.7 | 66,911,102 | 98.8 | 35.8 | 97.6 |

**Table S3.** DEGs Identified and Pathway Representation**.**

| Group | Total DEGs (Adj. P<0.05) | | Mapped in Metascape | Analysis-ready molecules in IPA | |
|---|---|---|---|---|---|
| **Comparison** | **DEGs** | **# up/# down** | **DEGs** | **DEGs** | **# up/# down** |
| Sham LV | 853 | 545/308 | 818 | 817 | 524/293 |
| 5/6Nx LV | 162 | 71/91 | 154 | 150 | 67/83 |
| Sham Kidney | 637 | 382/255 | 594 | 589 | 358/231 |
| 5/6Nx Kidney | 33 | 28/5 | 33 | 33 | 28/5 |

**Table S4.**

Top 15 Up- and Down-Regulated Genes with PD in LV from Sham Rats

| Gene Symbol | Change with PD | log2 Fold-Change | *P*-value | Adjusted *P*-value |
|---|---|---|---|---|
| *LOC100909700* | Up | 6.651754477 | 1.88E-11 | 1.63E-08 |
| *Cd177* | Up | 5.422109368 | 1.65E-10 | 1.22E-07 |
| *Reg3g* | Up | 3.203950967 | 6.38E-10 | 3.98E-07 |
| *Ifitm6* | Up | 3.084994373 | 4.71E-06 | 0.000638401 |
| *Robo3* | Up | 2.947755367 | 0.000853505 | 0.024651624 |
| *Cxcl2* | Up | 2.920479466 | 0.000871717 | 0.025065884 |
| *Reg3b* | Up | 2.866406019 | 2.90E-05 | 0.002319102 |
| *Rn60_4_1096.3* | Up | 2.807480716 | 0.000447207 | 0.015503291 |
| *Ptk6* | Up | 2.685344471 | 1.29E-05 | 0.001318059 |
| *Pabpn1l* | Up | 2.572217384 | 0.001135121 | 0.02986871 |
| *Cyp26b1* | Up | 2.522075951 | 6.10E-19 | 2.38E-15 |
| *Mt1m* | Up | 2.273301363 | 0.000100113 | 0.005398768 |
| *Mmp9* | Up | 2.268370159 | 1.07E-21 | 1.67E-17 |
| *Il1r2* | Up | 2.207220115 | 6.48E-05 | 0.004157467 |
| *Serpinb1a* | Up | 2.158464816 | 2.92E-15 | 7.59E-12 |
| *Cenpf* | Down | -1.296115381 | 2.73E-06 | 0.000435777 |
| *Ckap2l* | Down | -1.302636492 | 0.001669431 | 0.037489896 |
| *Scml2* | Down | -1.343345729 | 3.31E-05 | 0.002543971 |
| *Ddn* | Down | -1.347955862 | 5.67E-05 | 0.003774483 |
| *Pbk* | Down | -1.359405600 | 9.05E-06 | 0.00101906 |
| *Cep55* | Down | -1.373260127 | 6.60E-05 | 0.004179387 |
| *Shcbp1* | Down | -1.432611473 | 3.02E-05 | 0.002389574 |
| *Rnf43* | Down | -1.459947969 | 0.000866155 | 0.024951985 |
| *Bub1b* | Down | -1.527495558 | 5.81E-05 | 0.003836342 |
| *Mpv17l* | Down | -1.540323103 | 0.001692229 | 0.037635221 |
| *Ccna2* | Down | -1.543487140 | 0.002297518 | 0.044926998 |
| *Fam64a* | Down | -1.621052197 | 1.20E-05 | 0.00124169 |
| *Dtl* | Down | -1.635580634 | 0.000804232 | 0.023648977 |
| *Lrtm2* | Down | -1.949160044 | 2.60E-05 | 0.002169422 |
| *Depdc1* | Down | -3.785517324 | 0.001088186 | 0.028990381 |

**Table S5.**

Top 15 Up- and Down-Regulated Genes with PD in LV from 5/6Nx Rats

| Gene Symbol | Change with PD | log2 Fold-Change | *P*-value | Adjusted *P*-value |
|---|---|---|---|---|
| *LOC100909700* | Up | 4.475860551 | 2.42E-08 | 3.74E-05 |
| *Cd177* | Up | 4.034572294 | 5.17E-08 | 5.97E-05 |
| *Fgf23* | Up | 3.732680013 | 7.50E-05 | 0.014096 |
| *Selp* | Up | 3.220910092 | 5.40E-08 | 5.97E-05 |
| *Olfm4* | Up | 3.055003082 | 1.18E-07 | 0.000122 |
| *Igf2bp3* | Up | 2.219375396 | 0.000314 | 0.036499 |
| *Mmp9* | Up | 2.033977314 | 8.27E-19 | 1.28E-14 |
| *Vcam1* | Up | 1.897284975 | 4.33E-10 | 1.64E-06 |
| *Sele* | Up | 1.745493885 | 7.46E-06 | 0.003388 |
| *Ifitm6* | Up | 1.711440695 | 1.77E-07 | 0.000152 |
| *Mmp8* | Up | 1.506758746 | 6.63E-12 | 5.12E-08 |
| *Nsg2* | Up | 1.369463081 | 0.000224 | 0.028863 |
| *Retnlg* | Up | 1.333721187 | 0.000197 | 0.026902 |
| *Npas2* | Up | 1.241918166 | 4.25E-05 | 0.009662 |
| *S100a8* | Up | 1.236059480 | 2.13E-05 | 0.006331 |
| *Colec11* | Down | -1.136825017 | 1.25E-06 | 0.000964 |
| *Slc16a12* | Down | -1.162031228 | 1.81E-08 | 3.11E-05 |
| *Cpeb1* | Down | -1.203362854 | 3.21E-06 | 0.001658 |
| *Etv4* | Down | -1.217448947 | 1.04E-05 | 0.003836 |
| *Cdh24* | Down | -1.218643373 | 0.000291 | 0.034532 |
| *Xkr7* | Down | -1.276482749 | 8.73E-05 | 0.015258 |
| *Sypl2* | Down | -1.390912324 | 6.59E-05 | 0.012746 |
| *Ltbp2* | Down | -1.551124742 | 1.11E-05 | 0.003836 |
| *Wisp2* | Down | -1.813290670 | 5.17E-09 | 1.14E-05 |
| *Thbs4* | Down | -1.939153829 | 6.46E-05 | 0.012701 |
| *Crtac1* | Down | -2.363046468 | 0.000144 | 0.021584 |
| *Pacsin1* | Down | -2.507400727 | 1.42E-05 | 0.00455 |
| *LOC102552055* | Down | -2.557923815 | 6.49E-05 | 0.012701 |
| *Robo2* | Down | -2.726297447 | 0.000198 | 0.026902 |
| *Fcrl2* | Down | -2.946470679 | 2.47E-06 | 0.001554 |

**Table S6.**

Top Up- and Down-Regulated Genes with PD in Kidney from Sham Rats

| Gene Symbol | Change with PD | log2 Fold-Change | *P*-value | Adjusted *P*-value |
|---|---|---|---|---|
| *S100a8* | Up | 2.330891777 | 2.83E-13 | 2.19E-09 |
| *Mmp8* | Up | 2.099370352 | 1.43E-09 | 1.85E-06 |
| *Cyp26b1* | Up | 2.040648021 | 1.09E-10 | 2.01E-07 |
| *Tnfsf8* | Up | 1.96E+00 | 4.68E-05 | 0.004806681 |
| *S100a9* | Up | 1.861118235 | 2.17E-10 | 3.06E-07 |
| *Il1b* | Up | 1.668531961 | 4.23E-08 | 3.65E-05 |
| *Cyp26a1* | Up | 1.586276617 | 7.63E-05 | 0.00657641 |
| *Ptx3* | Up | 1.561920436 | 0.001420995 | 0.04088076 |
| *Mcemp1* | Up | 1.557191803 | 0.000341001 | 0.017120696 |
| *Mzb1* | Up | 1.545758994 | 0.001255218 | 0.03817379 |
| *C7* | Up | 1.540164191 | 0.001033518 | 0.034263202 |
| *Scara5* | Up | 1.532110627 | 2.31E-05 | 0.003102917 |
| *Msr1* | Up | 1.523238047 | 2.85E-05 | 0.003486577 |
| *Lum* | Up | 1.52E+00 | 0.001528201 | 0.042950203 |
| *Dmbt1* | Up | 1.497369752 | 1.26E-12 | 6.52E-09 |
| *Zyg11a* | Down | -1.142678572 | 8.02E-05 | 0.006790533 |
| *Scn8a* | Down | -1.168516279 | 1.25E-15 | 1.93E-11 |
| *Ccdc122* | Down | -1.22E+00 | 0.000192257 | 0.011651079 |
| *LOC680227* | Down | -1.221538087 | 0.000366826 | 0.018066494 |
| *Zfp136* | Down | -1.296222097 | 2.86E-12 | 8.87E-09 |
| *Polq* | Down | -1.37179719 | 0.000471939 | 0.02172599 |
| *Klrb1c* | Down | -1.42E+00 | 2.51E-05 | 0.003250753 |
| *Cpne4* | Down | -1.443362145 | 0.001691084 | 0.044897541 |
| *Best3* | Down | -1.512022714 | 7.31E-05 | 0.006422769 |
| *LOC100362814* | Down | -1.57E+00 | 5.89E-05 | 0.005506808 |
| *Htr5b* | Down | -1.627657935 | 0.000270738 | 0.014789528 |
| *Zbtb16* | Down | -1.93E+00 | 2.33E-06 | 0.00075316 |
| *Cyp8b1* | Down | -1.979362524 | 5.10E-06 | 0.00125086 |
| *Lrrtm3* | Down | -2.047841225 | 0.000640653 | 0.026283894 |
| *Rn60_20_0047.3* | Down | -8.45E+00 | 1.53E-05 | 0.002302402 |

**Table S7.**

Top Up- and Down-Regulated Genes with PD in Kidney from 5/6Nx Rats

| Gene Symbol | Change with PD | log2 Fold-Change | *P*-value | Adjusted *P*-value |
|---|---|---|---|---|
| *Fpr1* | Up | 5.311876280 | 1E-06 | 0.0018 |
| *Ptx3* | Up | 3.442213552 | 2E-06 | 0.0027 |
| *Mmp8* | Up | 3.065404366 | 2E-14 | 4E-10 |
| *Retnlg* | Up | 2.901578657 | 3E-10 | 1E-06 |
| *S100a8* | Up | 2.790976959 | 2E-13 | 1E-09 |
| *Bmper* | Up | 2.339630912 | 7E-06 | 0.0081 |
| *S100a9* | Up | 2.240599297 | 4E-10 | 1E-06 |
| *Dhrs9* | Up | 2.175648528 | 3E-10 | 1E-06 |
| *Pla2g2a* | Up | 2.028325853 | 1E-06 | 0.0021 |
| *Cxcl1* | Up | 1.786323824 | 3E-05 | 0.0201 |
| *Cd163* | Up | 1.685650985 | 1E-05 | 0.0129 |
| *Arntl* | Up | 1.478656030 | 3E-12 | 2E-08 |
| *Cxcr2* | Up | 1.447948533 | 1E-05 | 0.0129 |
| *RGD1559482* | Up | 1.418828454 | 4E-05 | 0.0253 |
| *Npas2* | Up | 1.219766060 | 2E-09 | 3E-06 |
| *Fus* | Down | -0.317631213 | 9E-08 | 0.0002 |
| *Osbpl6* | Down | -0.507636031 | 7E-05 | 0.0422 |
| *Cry2* | Down | -0.554344033 | 1E-05 | 0.0129 |
| *Tmprss3* | Down | -0.866948722 | 7E-06 | 0.0081 |
| *Per3* | Down | -0.973956038 | 7E-05 | 0.0405 |

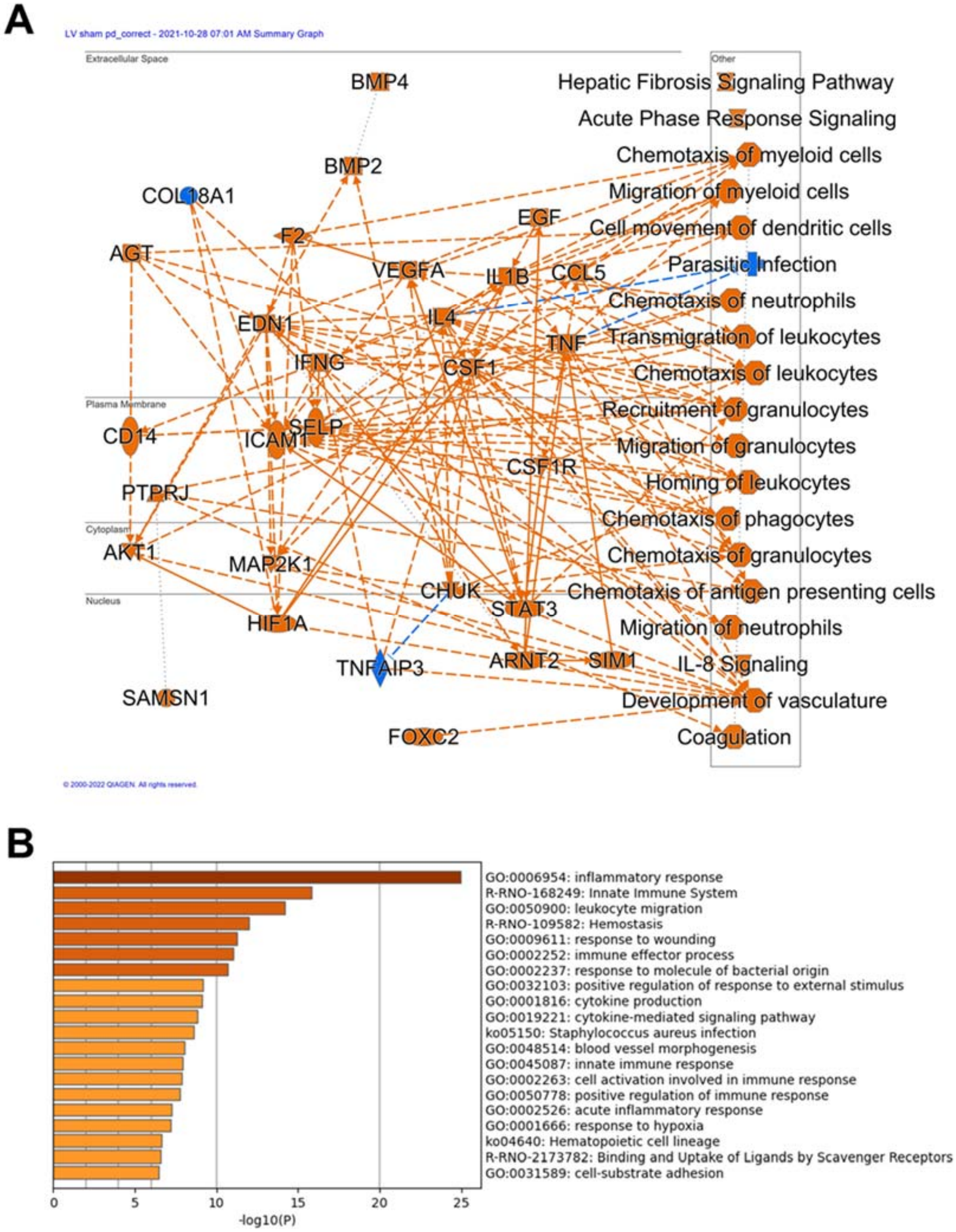


**Figure S1.** Pathway analysis of DEGs in LV of Sham-operated rats receiving PD vs. Controls. A) IPA "Graphical Summary" in "Subcellular" view. Nodes and lines are indicated as activated (orange) or inhibited (blue) based on expression differences. B) Metascape generated enriched functional terms ranked by -log10(P).

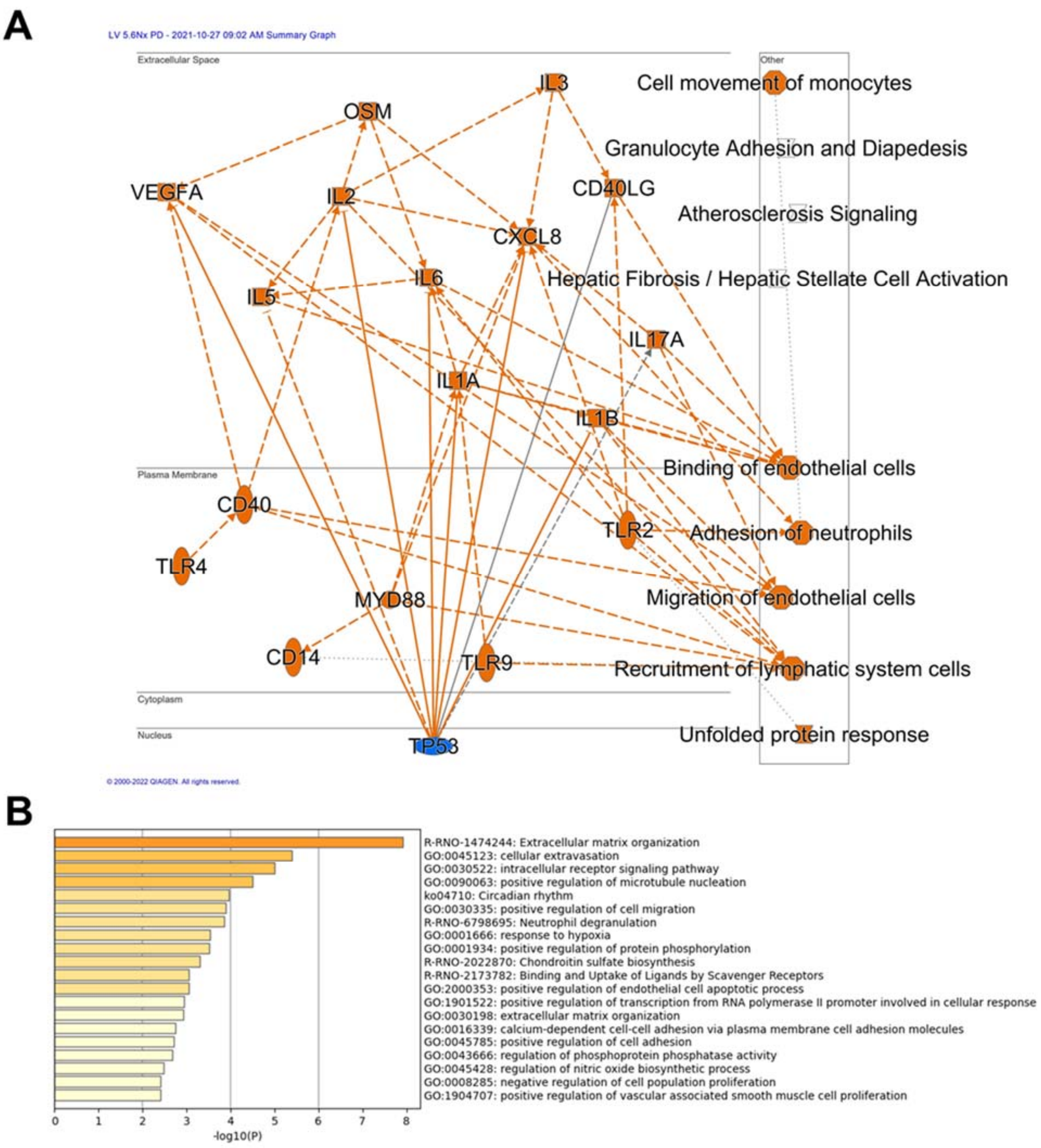


**Figure S2.** Pathway analysis of DEGs in LV of 5/6Nx animals receiving PD vs. Controls. A) IPA “Graphical Summary” in “Subcellular” view. Nodes and lines are indicated as activated (orange) or inhibited (blue) based on expression differences. B) Metascape generated enriched functional terms ranked by -log10(P).

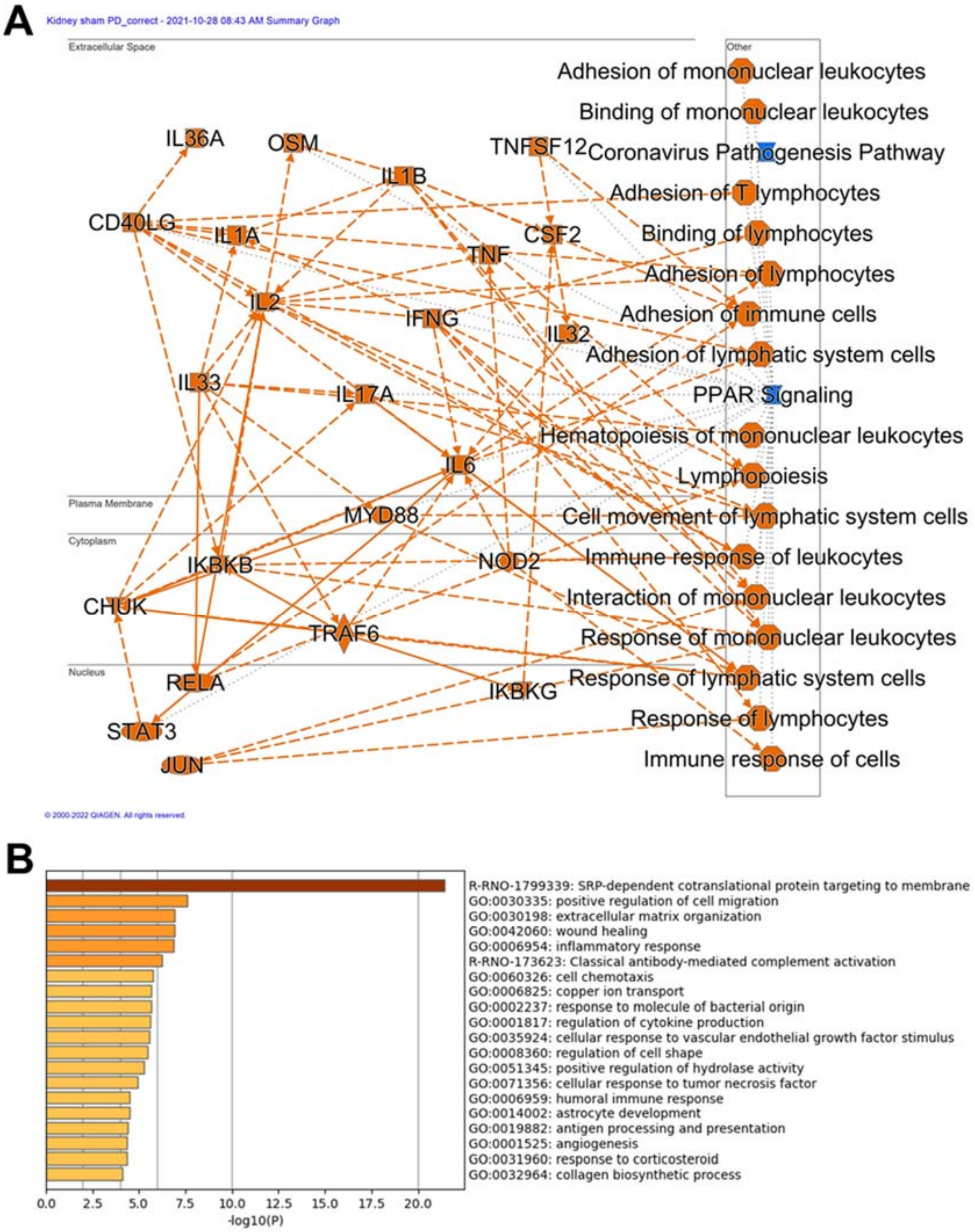


**Figure S3.** Pathway analysis of DEGs in kidney of Sham animals receiving PD vs. Controls. A) IPA "Graphical Summary" in "Subcellular" view. Nodes and lines are indicated as activated (orange) or inhibited (blue) based on expression differences. B) Metascape generated enriched functional terms ranked by -log10(P).

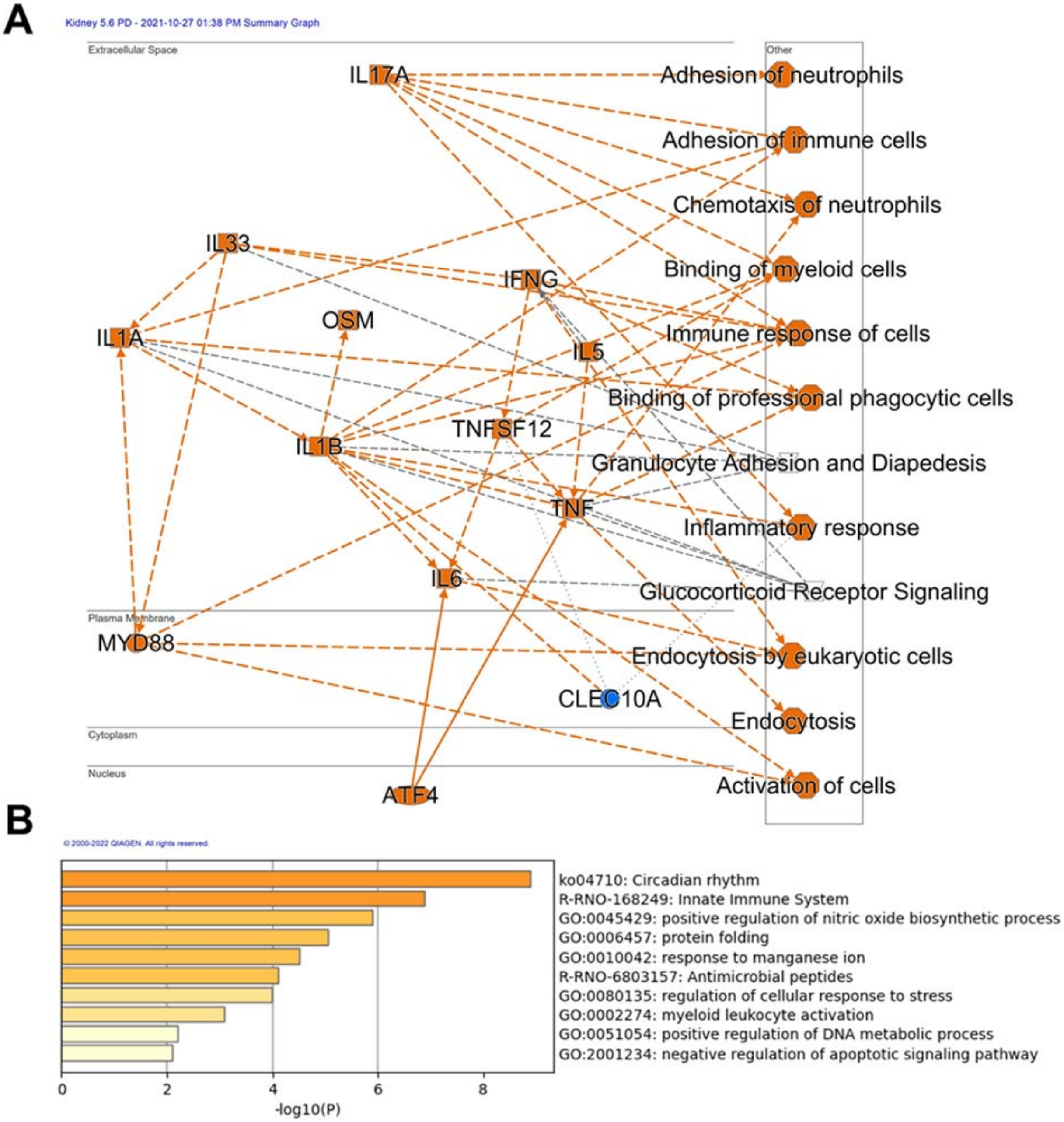


**Figure S4.** Pathway analysis of DEGs in kidney of 5/6Nx animals receiving PD vs. Controls. A) IPA "Graphical Summary" in "Subcellular" view. Nodes and lines are indicated as activated (orange) or inhibited (blue) based on expression differences. B) Metascape generated enriched functional terms ranked by -log10(P).

## Supplemental References

1. Krueger, F. *Trim Galore*. [cited 2021; Version 0.5.6:[Available from: https://www.bioinformatics.babraham.ac.uk/projects/trim_galore/.
2. Kim, D., et al., *Graph-based genome alignment and genotyping with HISAT2 and HISAT-genotype.* Nat Biotechnol, 2019. **37**(8): p. 907-915.
3. Kovaka, S., et al., *Transcriptome assembly from long-read RNA-seq alignments with StringTie2.* Genome Biol, 2019. **20**(1): p. 278.
4. Love, M.I., W. Huber, and S. Anders, *Moderated estimation of fold change and dispersion for RNA-seq data with DESeq2.* Genome Biol, 2014. **15**(12): p. 550.
5. Zhou, Y., et al., *Metascape provides a biologist-oriented resource for the analysis of systems-level datasets.* Nat Commun, 2019. **10**(1): p. 1523.
